# Experimental Infection of Voles with *Francisella tularensis* Indicates Their Amplification Role in Tularemia Outbreaks

**DOI:** 10.1371/journal.pone.0108864

**Published:** 2014-10-01

**Authors:** Heidi Rossow, Kristian M. Forbes, Eveliina Tarkka, Paula M. Kinnunen, Heidi Hemmilä, Otso Huitu, Simo Nikkari, Heikki Henttonen, Anja Kipar, Olli Vapalahti

**Affiliations:** 1 Department of Veterinary Biosciences, Faculty of Veterinary Medicine, University of Helsinki, Helsinki, Finland; 2 Department of Biological and Environmental Science, University of Jyväskylä, Jyväskylä, Finland; 3 Division of Clinical Microbiology, Helsinki University Hospital Laboratory (HUSLAB), Helsinki, Finland; 4 Centre for Biothreat Preparedness, Centre for Military Medicine, Finnish Defence Forces, Helsinki, Finland; 5 Finnish Forest Research Institute, Vantaa, Finland; 6 Finnish Centre for Laboratory Animal Pathology, Faculty of Veterinary Medicine, University of Helsinki, Helsinki, Finland; 7 School of Veterinary Science and Department of Infection Biology, Institute of Global Health, University of Liverpool, Liverpool, United Kingdom; 8 Department of Virology, Haartman Institute, University of Helsinki, Helsinki, Finland; The Johns Hopkins University School of Medicine, United States of America

## Abstract

Tularemia outbreaks in humans have been linked to fluctuations in rodent population density, but the mode of bacterial maintenance in nature is unclear. Here we report on an experiment to investigate the pathogenesis of *Francisella tularensis* infection in wild rodents, and thereby assess their potential to spread the bacterium. We infected 20 field voles *(Microtus agrestis)* and 12 bank voles (*Myodes glareolus*) with a strain of *F. tularensis* ssp. *holarctica* isolated from a human patient. Upon euthanasia or death, voles were necropsied and specimens collected for histological assessment and identification of bacteria by immunohistology and PCR. Bacterial excretion and a rapid lethal clinical course with pathological changes consistent with bacteremia and tissue necrosis were observed in infected animals. The results support a role for voles as an amplification host of *F. tularensis*, as excreta and, in particular, carcasses with high bacterial burden could serve as a source for environmental contamination.

## Introduction

*Francisella tularensis* is a zoonotic intracellular bacterium that belongs to the *γ*-subclass of *Proteobacteria* [1], [2]. Two *F. tularensis* subspecies cause clinical infections in humans: *F. tularensis* subsp. *tularensis* (type A), which is almost exclusively found in North America, and *F. tularensis* subsp. *holarctica* (type B), which occurs throughout the Holarctic region [3]. In Finland, dozens to several hundreds of human tularemia cases are registered each year, and incidence rates show marked geographical variation between districts [4]. From 1996 to 2004, the cumulative incidence of human tularemia in Finland was over 37 cases/100,000 inhabitants, which is the highest of all EU member states [5]. Meanwhile, a series of outbreaks has demonstrated the re-emergence of this disease in other European countries [6]–[8].

*F. tularensis* is renowned for its high infectivity and wide host range. The infectious dose for humans can be as low as 10 bacteria [9], and the bacterium has been isolated from numerous mammalian species, including rabbits, hares, voles and other rodents [10]–[13], and detected from natural waters and mud, and from mosquito larvae collected in endemic areas [14], [15]. It is very likely that *F. tularensis* persists in natural waters, possibly in aquatic protozoa [16].

Humans become infected with *F. tularensis* through arthropod bites, direct contact with infected animals, inhalation of infective aerosols, or ingestion of contaminated food or water [4], [9]. Clinical manifestations depend mainly on the infection route, and the disease severity depends on the infecting subspecies and strain [17]. After an incubation period of approximately 3–5 days (range: 1–14 days), non-specific influenza-like symptoms, especially fever, chills and headache, arise usually with rapid onset [2], [9], [17], [18]. Infection through the skin results in ulceroglandular tularemia, while infection via the mucous membranes induces ulceroglandular, glandular, oculoglandular, or oropharyngeal tularemia [2], [17]. In Fennoscandia, where the bacterium is transmitted mainly through mosquito bites [4], [19], the ulceroglandular form is most common [4]. Inhalation of aerosolized *F. tularensis* causes pulmonary tularemia, the most severe form of the disease [20]–[22].

Tularemia outbreaks in humans have been linked to high rodent densities [7], [18], [23]–[25], and exposure to rodents or their droppings is suspected as the infection source in a large outbreak in Kosovo [24], [25]. However, the precise role of rodents in bacterial maintenance, and the nature of their association with human disease have remained unclear. In Finland, the field vole *(Microtus agrestis)* and bank vole (*Myodes glareolus*) are the dominant rodent species [26], and hence the most plausible hosts for *F. tularensis*. Indeed, we have recently detected the bacterium in screening of wild field voles in Finland [27]. Here we report on an experiment to evaluate the pathogenicity of *F. tularensis* for these species, in order to further elucidate factors affecting their association with human disease outbreaks.

## Materials and Methods

### Ethics

Experimental procedures and facilities were approved by the Finnish Animal Experiment Board (Permit ESAVI/6162/04.10.03/2012), which followed the Finnish legislation for animal experiments. All efforts were made to minimize animal suffering.

### Naturally infected animals

Three naturally *F. tularensis-*infected, PCR-positive adult field voles, trapped as part of a screening project in the Konnevesi area in Central Finland [27], were evaluated for the presence of bacteria in tissues and associated pathological changes as a reference for the experimental infection study. Tissue specimens from lungs, liver and kidneys were collected from these animals and frozen at −20°C. Samples were later thawed and fixed in 10% buffered formalin for histopathological and immunohistological examination.

### Animals for experimental infections

The experimental infections were conducted on visibly healthy adult (>8 weeks of age) field and bank voles. These animals were laboratory-born at the Finnish Forest Research Institute, Suonenjoki station, and were the progeny of wild voles captured in the surrounding area.

For the experimental infections, voles were transferred to the biosafety level 3 laboratory of the Faculty of Veterinary Medicine, University of Helsinki, Finland, where they were housed in individually ventilated and HEPA-filtered isolation cages (Isocage Unit, Tecniplast, Italy). Wood shavings covered the cage floor, and a cardboard roll was supplied for additional cover. Water and rodent pellets (22.5% crude protein, 5% crude fat, 4.5% crude fiber and 6.5% crude ash) were supplied *ad libitum*, and voles were given a slice of fresh apple every 1–2 days. Voles were placed into the cages three days prior to experimental infections.

### Bacteriology

A strain of *F. tularensis,* which had originally been isolated from a cutaneous ulcer of a 49-year-old woman, identified as ssp. *holarctica* by 16S rRNA gene sequencing, was used for the experimental infections. Bacteria were cultured on chocolate agar plates and incubated at +35°C in 5% CO_2_ for five days. MacFarland 1.0 suspension was prepared in sterile isotonic saline and diluted in ten-fold series to approximately 1000 colony-forming units (cfu)/ml. The actual concentration was determined by plate counting in each experiment. The diluted suspension was kept on ice and used for inoculations within 1–2 h of preparation. The viable count of *F. tularensis* in the remaining dilution was similar to that of the fresh dilution.

### Experimental infections

#### Pilot study

A pilot study was conducted to identify a bacterial delivery route and dose that best mimic natural infections in voles, and to gather information on the incubation period and clinical course of infection. For this, two field voles were allocated to each of 4 dose/route combinations (total n = 8): either 120 (low dose) or 1,200 (high dose) cfu of *Francisella tularensis* ssp. *holarctica* (diluted in 100 µl of sterile isotonic saline), and either intranasal (i.n.) or subcutaneous (s.c.) delivery route. Experimental infections were conducted under brief isoflurane anesthesia, and s.c. injections were delivered between the shoulder blades. One further vole served as an uninfected control.

The animals were checked twice daily for signs of illness or death, and immediately euthanized if they exhibited signs of illness. After 9 days, all remaining voles were euthanized via cervical dislocation under isoflurane anesthesia. A full *post mortem* examination was performed immediately after death or when the voles were found dead, and samples from the spleen, lung, liver, and kidney were aseptically collected and frozen at −80°C for PCR analysis. In addition, samples of heart, lungs, liver, kidneys, spleen, mesenteric and mediastinal lymph nodes, brain, and inoculation sites (skin, nose) were fixed in 10% buffered formalin, for histological and immunohistological assessment.

#### Main study

For the main experiment, 12 field voles and 12 bank voles were injected s.c. with a 100 µl suspension containing 70 cfu of *F. tularensis* ssp. *holarctica* in sterile isotonic saline. Three randomly selected animals of each species served as non-infected controls and were injected with 100 µl sterile isotonic saline alone. Voles were checked twice daily for signs of illness and death. Three infected voles of each species were electively euthanized on days 1 and 3 post infection (p.i). The remaining voles were euthanized by cervical dislocation under isoflurane anesthesia if symptomatic. Animals were necropsied immediately after death, and urine, feces, spleen, and kidney samples were aseptically collected and frozen at −80°C for PCR analysis. Tissue specimens from lungs, liver, spleen, bone marrow, kidneys, stomach, duodenum, jejunum, colon, and the inoculation site were fixed in 10% buffered formalin for histological and immunohistological assessment.

### Histology and immunohistology

Fornalin-fixed tissue specimens from all animals were trimmed and routinely paraffin wax embedded. Sections (3–5 µm) were prepared and stained with hematoxylin-eosin (HE) or used for immunohistology (IH). IH was performed using a mouse monoclonal antibody against *F. tularensis* LPS (clone T14; Meridian Life Sciences, Memphis, USA) and the horseradish peroxidase method (Envision; Dako, Glostrup, Denmark) with diaminobenzidine as chromogen, after antigen retrieval with citrate buffer (pH 6.0) microwave pretreatment.

### DNA extraction and PCR analyses

DNA was extracted from vole tissue samples and excreta using commercial kits. The Wizard Genomic DNA Purification Kit (Promega, Madison, USA) was used for spleen and kidney samples, following the protocol for animal tissue. The QIAamp DNA Stool kit (Qiagen, Hilden, Germany) was employed for fecal samples (20 mg feces+160 µl phosphate-buffered saline). From urine samples (24.5–140 µl), DNA was extracted with the QIAamp Viral RNA Mini kit (Qiagen, Hilden, Germany), using the protocol for purification of cellular, bacterial, or viral DNA from urine. Each sample batch contained water as a negative control. DNA concentration and purity were determined with the Nanodrop ND-1000 spectrophotometer (Thermo Scientific, Wilmington, DE, USA).

The DNA samples were subjected to a modified semi-quantitative real-time PCR assay (qPCR) targeting the 23 kDa gene of *F. tularensis* (27, 28). All PCRs were run in duplicate with an ABI 7500 instrument (Applied Biosystems, Foster City, CA, USA). DNA from tissue samples was analyzed using 1∶100 dilutions, and for urine and fecal samples, three 10-fold (undiluted, 1∶10, 1∶100) dilutions were examined. The PCR assay included an internal positive inhibition control, water as negative non-template control, and *F. tularensis* LVS control strain DNA as positive control. The amount of *F. tularensis* bacteria in each sample was estimated based on genomic equivalents (GE). To enable comparison of *F. tularensis* amounts in tissues of experimentally versus naturally infected voles, we also calculated the GE amount in relation to the estimated number of cells in tissue samples [27].

## Results

### Naturally infected wild field voles

**Figure 1:**
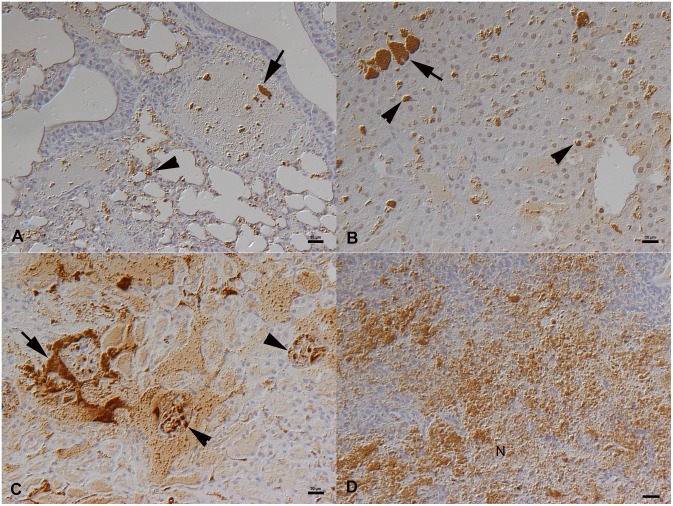
Naturally *F. tularensis sp. holartica* infected field vole that had been trapped and euthanized [27]. **A**. Lung with bacterial clumps in vessel lumina (arrow) and bacterial aggregates in capillaries (arrowhead). **B**. Liver with bacterial clumps in sinusoids (arrow) and smaller aggregates within Kupffer cells (arrowheads). **C**. Kidney with bacterial aggregates in larger vessels (arrow) and glomerular capillaries (arrowheads). **D**. Spleen with abundant bacterial clumps, in association with necrosis (N), in the red pulp. Horseradish peroxidase method, Papanicolaou’s hematoxylin counterstain. Bars = 20 µm.

In the three *F. tularensis*-infected wild field voles [27], bacteremia was confirmed by histology and immunohistology. Bacteria were found as aggregates within vessels and capillaries, specifically also in liver sinusoids and renal glomerular capillaries (Figure 1A–D). They were abundant in the splenic red pulp where they were associated with extensive necrosis (Figure 1D). In addition, bacteria were identified within macrophages in the liver (i.e. Kupffer cells: Figure 1B) and the splenic red pulp. In the livers, individual necrotic hepatocytes were also seen.

### Pilot study in field voles

A pilot study was conducted on field voles to evaluate different infection routes (s.c. and i.n.) and doses (high and low dose). All voles remained asymptomatic during the first four days after infection. On day 5 p.i., four infected voles (two low dose s.c., one high dose i.n., and one high dose s.c.) were found dead, and another animal (high dose s.c.) was euthanized due to general malaise. On day 6 p.i., one symptomatic vole (high dose i.n.) was euthanized. Both low dose i.n. infected voles survived until day 9 p.i., when one was found dead and the other, which had remained asymptomatic, as well as the uninfected control animal, were electively euthanized at the scheduled end of the experiment.

**Figure 2:**
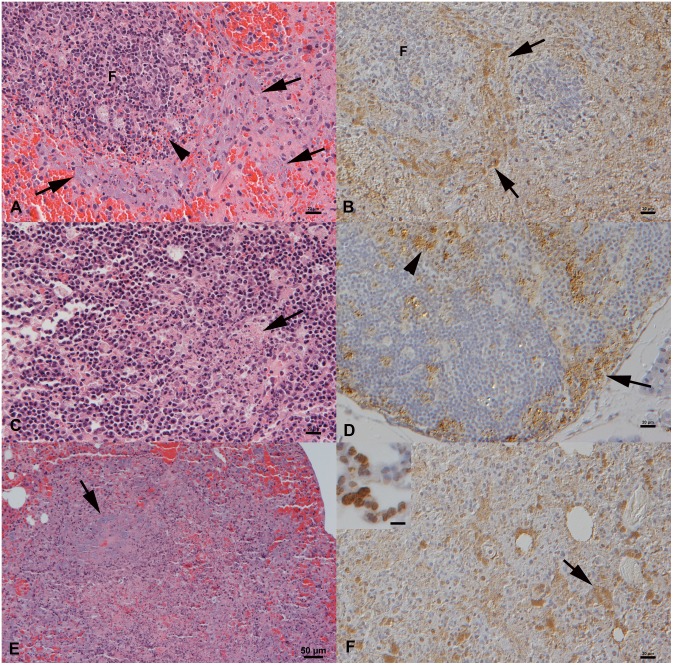
Pilot study, field voles that had died from or were euthanized after experimental *F. tularensis sp. holartica* infection. **A, B**. Spleen from an animal found dead on day 5 after subcutaneous (s.c.) infection with a low dose (LD). Clumps of bacteria are found cell-free and within (degenerating) macrophages in the red pulp, often surrounding follicles (F). Numerous lymphocytes in follicles undergo apoptosis (A: arrowhead). **C, D**. Mesenteric lymph node from an animal found dead on day 5 after s.c. infection with a high dose (HD). C. Cortex with focal area of necrosis (arrow). **D**. Clumps of bacteria are present within the sinuses (arrow) and in areas of necrosis (arrowhead). **E, F**. Lung from a vole found dead on day 9 after HD intranasal infection. Focal area of extensive necrosis with large aggregates of bacteria (arrows). Inset in F: Lung from a vole that was euthanized on day 5 after HD s.c. infection. Bacteria are found within circulating leukocytes in lung capillaries. A, C, E. HE stain, B, D, F. Horseradish peroxidase method, Papanicolaou’s hematoxylin counterstain. Bars = 20 µm (A–D, F), 50 µm (E), 10 µm (Inset F).

The *post mortem* examination did not reveal any significant gross changes. Histology confirmed severe bacteremia in all but the electively euthanized low dose i.n. infected vole and the control animal, with bacterial aggregates in vessels in all examined organs and in the cardiac chambers. The pathological changes were very similar to those seen in the naturally infected voles and are typical for tularemia in other species [10], [13], such as extensive splenic and lymph node necrosis with abundant cell-free bacteria (Figure 2A–D). Two i.n. infected voles (one high dose and one low dose) also showed a multifocal extensive necrotizing pneumonia with abundant bacteria both cell-free and in macrophages (Figure 2E, F), features not seen in the naturally infected voles. This indicates direct aerosol infection of the lung and subsequent bacteremia, in particular since the animals exhibited neither histological changes nor bacteria in the nasal cavity. Bacterial loads in organs did not substantially vary in relation to the route and dose of infection and were generally high in all tissues of symptomatic animals. In the infected vole that had remained asymptomatic, systemic infection was confirmed by PCR, albeit with low bacterial organ loads and without any histological changes or IH evidence of bacteria in any examined tissue.

### Main experimental study in field and bank voles

For the main study, a low dose delivered via s.c. injection was chosen, as the pilot study demonstrated it to best mimic the natural infection in voles.

**Figure 3:**
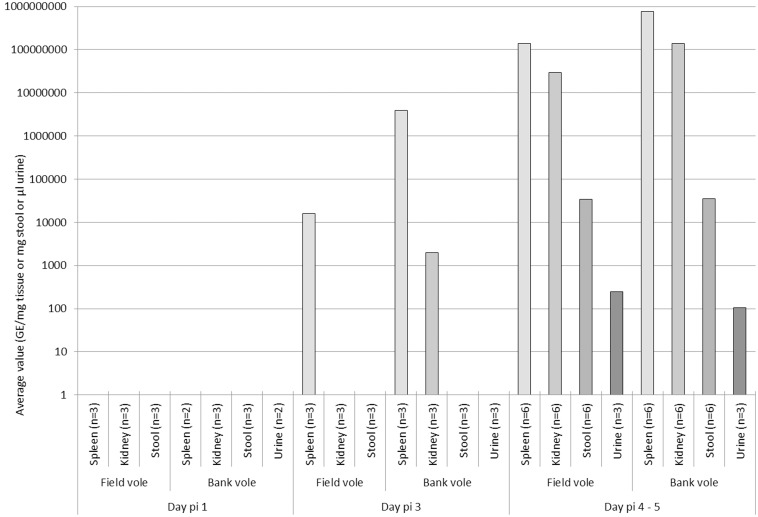
Quantification of *F. tularensis* DNA in spleen, kidney, faeces and urine of infected voles by day post infection determined using real-time PCR targeting the bacterial 23 kDa gene of *F. tularensis* [27], [28]. Samples were collected at the following time points: day 1 post infection (p.i.), day 3 p.i. and days 4–5 p.i.

All animals that were sacrificed on days 1 and 3 p.i. (three field voles and three bank voles at each time point) had been asymptomatic and did not exhibit any significant gross changes. On day 1 p.i., PCR did not detect *F. tularensis* DNA in spleen, kidney, feces or urine (Figure 3), and IH did not identify bacteria in any tissue (Table 1). Histological changes were restricted to the injection site, where focal interstitial hemorrhage was generally seen. In one bank vole a focal macrophage aggregate was found in the adipose tissue of the inoculation site, and IH identified a few bacteria within the macrophages.

**Table 1:** Detection of *Francisella tularensis* in organs and excretions of experimentally infected voles.

	No. PCR^a^- and IH^b^ -positive/No. examined							
Species and specimens	Day 1		Day 3		Day 4		Day 5	
*Microtus agrestis*,field vole	PCR	IH	PCR	IH	PCR	IH	PCR	IH
Spleen	0/3	0/3	3/3	1/2	1/1	1/1	5/5	5/5
Kidney	0/3	0/3	0/3	0/3	1/1	1/1	5/5	5/5
Feces	0/3		0/3		0/1		5/5	
Urine	NA		NA		1/1		2/2	
***Myodes glareolus*** **,** **bank vole**								
Spleen	0/3	0/3	3/3	3/3			6/6	6/6
Kidney	0/3	0/3	2/3	2/3			6/6	6/6
Feces	0/3		0/3				6/6	
Urine	0/3		0/3				3/3	

a real-time PCR targeting the bacterial 23 kDa gene of *F. tularensis* [27], [28].

b IH, Immunohistology using a mouse monoclonal antibody against *F. tularensis* lipopolysaccharide (clone T14; IgG3).

No, number; PCR, polymerase chain reaction; IH, immunohistology; NA, not available.

Denominators represent the total amount of screened animals.

**Figure 4:**
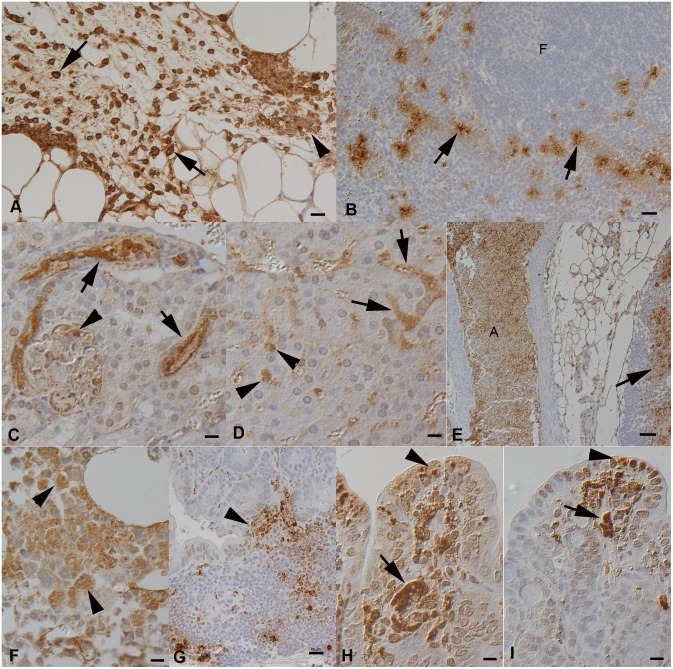
Main study, voles after subcutaneous infection with *F. tularensis sp. holartica*. **A, B**. Day 3 post infection (p.i.). **A**. Field vole, injection site in the subcutis with moderate pyogranulomatous inflammation. Bacteria are present within leukocytes (arrows) and as cell-free aggregates (arrowhead). **B**. Bank vole, spleen. In the red pulp, in particular surrounding follicles (F) and T cell zones, are aggregates of macrophages with intracellular bacteria (arrows). **C–I**. Day 5 post infection. **C**. Field vole, kidney. Bacteria form clumps in the lumen of interstitial veins (arrows) and glomerular tufts (arrowhead). **D**. Field vole, liver. Bacteria form aggregates within sinusoids (arrows) and are present within Kupffer cells (arrowheads). **E**. Field vole, mesenteric lymph node and large artery (A). Cell-free bacteria fill the lumen of the artery and are present within necrotic areas in the lymph node (arrow). **F**. Bank vole, bone marrow. Bacteria are mainly found within mononuclear (myeloid) cells (arrowhead). **G**. Field vole, duodenum with Peyer’s patch, exhibiting bacteria within cells and cell free, also towards the mucosal surface (arrowhead). **H**. Field vole, jejunum. Bacterial aggregates fill capillaries (arrow) and are present within cells, also in the lamina epithelialis mucosae (arrowhead). **I**. Bank vole, colon. Bacterial aggregates fill capillaries (arrow) and are present within cells, also in the lamina epithelialis mucosae (arrowhead). Horseradish peroxidase method, Papanicolaou’s hematoxylin counterstain. Bars = 20 µm (A–D, G), 50 µm (E), 10 µm (F, H, I).

On day 3 p.i., a neutrophil-dominated inflammatory reaction with intracellular (macrophages, neutrophils) and cell-free bacteria was often seen at the inoculation site (Figure 4A). The spleen of all animals tested positive for *F. tularensis* DNA (Figure 3), and in all but one weakly PCR-positive spleen, IH identified variable amounts of bacteria within macrophages in the red pulp (Figure 4B, Table 1), confirming cell-associated bacteremia. This was not associated with distinct histological changes in the spleen. In two bank voles, the kidney was weakly PCR-positive, and IH identified some bacteria in glomerular capillaries, without other histological changes. The urine of both these animals was PCR-negative. IH also identified bacteria in the livers, as individual cells in sinuses, and identified patches of reactive hepatocytes. Some bacteria were found in capillaries in the lungs of the three bank voles, again without distinct histopathologic changes.

On day 4 p.i., one field vole displayed general malaise and was euthanized, and on day 5 p.i., the remaining 5 field voles and 6 bank voles died or were visibly symptomatic and euthanized. PCR demonstrated *F. tularensis* DNA in the urine and high *F. tularensis* loads in the spleens and kidneys of all animals (Figure 3); in voles euthanized on day 5 p.i., *F. tularensis* was also identified in feces (Figure 3, Table 1). Histology and IH confirmed these results and revealed features similar to those in the pilot study. The findings were similar in both species. In general, large bacterial aggregates were seen in the splenic red pulp and in capillaries in all examined organs. In the kidneys, bacteria were found in both glomerular and interstitial capillaries (Figure 4C). Apart from disseminated bacterial aggregates between hepatic cords, the liver carried bacteria within Kupffer cells and exhibited multifocal random hepatocellular necrosis (Figure 4D).

In the spleen, the red pulp was almost completely effaced due to necrosis (and loss) of cells, and the white pulp was markedly reduced, with extensive (follicular) apoptosis/necrosis and replacement by bacteria. Lymph nodes exhibited focal areas of necrosis with abundant bacteria (Figure 4E). In the bone marrow, bacteria were found within mononuclear cells (most consistent with macrophages) and sometimes cell free (Figure 4F), and there was extensive necrosis/apoptosis of myelopoietic cells. Examination of the gastrointestinal tract identified bacteria within capillaries in all compartments, within Peyer’s patches (Figure 4G) and occasionally also in intestinal epithelial cells in both the small and large intestine (Figure 4H, I). Some small macrophage aggregates with bacteria were found in the lamina propria mucosae. More extensive inflammatory infiltrates were restricted to inoculation sites, where variably extensive necrosis and neutrophil infiltration with masses of cell-free bacteria was seen.

Control voles remained asymptomatic and were euthanized on day 9, at the scheduled end of the experiment. They were negative for *F. tularensis* by PCR and IH and did not exhibit any histological changes.

## Discussion

The current study presents an experimental model that mimics natural *F. tularensis* ssp. *holarctica* infection of wild voles and demonstrates that both field voles and bank voles are highly susceptible to the bacterium. Infected animals died with bacteremia, following a rapid clinical course and generally with very high bacterial loads in organs. We showed that infected voles excrete *F. tularensis* in their urine and feces around the time of death. The bacterial burden in excreta was relatively low compared to the bacterial load in tissues, but since only a low dose is generally required for infection [9], feces and urine might be infective for other animals and humans. Furthermore, the course of infection could be different under natural conditions. Long-term infections and shedding of *F. tularensis* have been reported after oral infection [29], [30] and the oral route of infection should be studied in future. The presence of bacterial aggregates within the glomerular tufts in the kidneys and within mucosal vessels and between epithelial cells in the intestinal mucosa of animals by day 5 p.i. also indicates that *F. tularensis* is excreted in urine and feces at this stage. Excretion of *F. tularensis*, in addition to contamination from dead animals, might serve to transfer the bacteria into the environment, which could also include mosquito breeding sites. In support of this premise, *F. tularensis* has been demonstrated to survive in water for several weeks [31], [32]. The survival is supported by protozoa, which are commonly found in natural aquatic systems as part of their normal biofilms [16].

Outbreaks of airborne tularemia in humans are mainly linked to farm work and other outdoor activities [6], [8], [12], [22], [33], [34], for example exposure to hay dust has been associated with pneumonic tularemia [4]. This might be due to bacteria-containing aerosols originating from animal carcasses or excreta made airborne by agricultural machines. Similarly, Puumala hantavirus infection is acquired by inhalation from rodent excreta, and considerably more often by farmers [35]. *F. tularensis* has been shown to survive up to 192 days in the environment on straw and grain depending on the temperature of the surrounding air [36]. Survival is longest in winter conditions, as the amount of viable bacteria decreases with rising temperatures [36]. The enhanced survival of *F. tularensis* in cool temperatures might be one factor contributing to the high tularemia incidence in Fennoscandia.

Our analysis of the pathogenesis of tularemia indicates that the bacteria are taken up locally (i.e. at the inoculation site) by macrophages and neutrophils and then distributed throughout the body, to eventually accumulate in the blood. Accordingly, they were found both within monocytes and cell free in vessels of almost all organs, and led to necrosis of infected cells, resulting in extensive necrosis particularly in the lymphatic tissues (i.e. spleen and lymph nodes). Interestingly, apart from the inoculation site, this was not associated with an overt inflammatory response. Similar changes have been reported in hares, in which tularemia is mainly characterized by acute focal necrosis without cellular reaction in liver, spleen, and bone marrow [10]. Recently, *F. tularensis* infection even without lesions has been described in squirrels [37]. In our pilot study, two intranasally infected voles exhibited a necrotizing to granulomatous pneumonia, indicating direct infection of the lung (not via bacteremia). This kind of prominent change is typical for inhalational tularemia; severe necrotizing pneumonia has been demonstrated in monkeys [38] and mice [39] after *F. tularensis* spp. *tularensis* aerosol exposure. Necrotizing granulomatous inflammation is also seen in lung biopsies of human patients with pulmonary tularemia [40], [41].

In Fennoscandia, tularemia is primarily mosquito-transmitted, and large human outbreaks occur regularly [4], [19]. Mosquitoes have been shown experimentally to become persistently infected already as larvae and then transstadially through the developmental stages to adults, without evidence of *F. tularensis* replication, however [42]. It has been shown that *F. tularensis* multiplies in protozoa [16], but mammals are probably also needed as local amplifiers to facilitate the spread of the disease [42] e.g. through contaminated water and subsequently mosquitoes. In Sweden, a temporal link between outbreaks in humans and rodent density cycles has been reported during 1960s and 1970s [23]. Moreover, our recent survey of wild rodent species identified *F. tularensis* in wild field voles [27], and we show here that the massive bacteremia and pathological lesions after experimental infection are identical to those in naturally infected animals. Mosquitoes might also become infected by feeding on bacteremic voles and then perhaps directly transmit *F. tularensis* to humans and other susceptible hosts. It is also possible that *F. tularensis*, amongst other factors, contributes to the density crash of vole populations in certain areas, at which stage *F. tularensis* is released into the environment. This environmental contamination presumably also propagates the outbreak among voles. As our results show, infected dead voles can lead to heavy contamination of the environment and provide an explanation for the common association between rodent density and human tularemia incidence.

In summary, the fact that voles readily developed lethal tularemia, together with the severity and similarity of the lesions in both experimentally and naturally infected animals, suggest that long-term or latent infection of these species is unlikely, yet some reservation concerning the infection routes may be warranted. Instead, voles are likely to play a role as amplification hosts and lead to bacterial contamination of the local environment, and by this mechanism contribute to the incidence of human tularemia.
